# Altered functional connectivity of the marginal division in migraine: a resting-state fMRI study

**DOI:** 10.1186/s10194-016-0682-1

**Published:** 2016-09-26

**Authors:** Zhiye Chen, Xiaoyan Chen, Mengqi Liu, Shuangfeng Liu, Siyun Shu, Lin Ma, Shengyuan Yu

**Affiliations:** 1 Department of Radiology, Chinese PLA General Hospital, Beijing, 100853 China; 2 Department of Neurology, Chinese PLA General Hospital, Beijing, 100853 China; 3 Institute of Cognitive Neuroscience, South China Normal University, Guangzhou, 510631 China

**Keywords:** Migraine, Marginal division of the neostriatum, fMRI, Functional connectivity

## Abstract

**Background:**

The marginal division of neostriatum (MrD) is a flat, pan-shaped zone between the neostriatum and the globus pallidus, and previous documents demonstrated that it was involved in the modulation of pain. The aim of this study is to investigate the roles of the MrD of the human brain in the chronicization migraine using resting state functional magnetic resonance imaging (rs-fMRI).

**Methods:**

Conventional MRI, 3D structure images, and rs-fMRI were performed in 18 patients with episodic migraines (EM), 16 patients with chronic migraine (CM), 44 patients with medication overuse headache plus chronic migraine (MOH + CM), and 32 normal controls (NC). MrD was defined using manual delineation on structural images, and was selected as the seed to calculate the functional connectivity (FC).

**Results:**

Compared with the NC group, the decreased FC of MrD was observed in the EM and CM groups, and increased FC of MrD was demonstrated in all patient groups. Compared with the EM group, the decreased FC of MrD was revealed in the CM and MOH + CM groups, and the increased FC occurred only in the CM group. Increased FC of MrD alone was observed in the MOH + CM group compared with that in the CM group.

**Conclusion:**

This study confirmed the double neuromodulation network of MrD in pain modulation and migraine chronicization; however, the mechanism requires further investigation.

## Background

The marginal division of neostriatum (MrD) is a flat, pan-shaped zone between the neostriatum and the globus pallidus, consisting of spindle-shaped neurons and some special connections [1, 2]. This area was first discovered at the caudal border of the striatum and the surrounding areas on the rostral edge of the globus pallidus in rat brains using histochemical techniques in 1988 [1]. Gradually more relevant papers were published, some of which demonstrated that this region was rich in neurotransmitters [3–5] and might also play an important role in learning and memory [2, 6–8].

In addition, neurophysiological studies showed that MrD was involved in the modulation of pain due to nociceptive neurons localized exclusively in rat striatums [9, 10]. Substance P, an important neuropeptide in the MrD, plays a key role in learning and memory [11], which is also related to headache [12]. The rats with lesions of the MrD induced by kainic acid experienced altered perception of neuropathic pain behaviors. This may also be associated with the evident increased substance P in the ipsilateral spinal cord dorsal horn [13]. Mu opioid receptors (MORs) are localized in the MrD, and it is one member of the seven transmembrane family of G-protein coupled receptors, which may underlie pain and analgesia in the MrD of rat striatum [14]. To date, however, it has remained unknown whether and how MrD participates in pain modulation in the human brain in vivo.

Brain imaging and imaging analysis techniques can provide a possibility to explore and evaluate MrD in vivo in the human brain. A current study demonstrated that altered functional connectivity of MrD was shown in Alzheimer’s disease by using resting state functional MRI (rs-fMRI) [15]. In our previous studies, this method was used to investigate the age and gender effects of functional connectivity of MrD for the normal subjects [16, 17]. In clinical practice, the decreased functional connectivity of MrD was demonstrated in a patient with right extremity pain caused by a malacia lesion in the left putamen using rs-fMRI, which suggested that the MrD may contribute to the pain modulation [18].

The aim of this study is to investigate the roles of MrD in the chronicization of migraine using rs-fMRI. We hypothesized that MrD was involved in the pain modulation in headache, and that the decreased functional connectivity of MrD would generate the pain and might be the cause of migraine, while the increased functional connectivity of MrD would be complementary for pain and might compensate the dysfunction of MrD neuromodulation. To address this hypothesis, we obtained functional MR images for normal controls (NC), episodic migraine (EM) patients, chronic migraine (CM) patients, and medication overuse headache plus with chronic migraine (MOH + CM) patients. Firstly, the functional connectivity of MrD was performed with within-group analysis to explore the functional connectivity pattern of different subtypes of headache. Secondly, the between-group analysis was performed between the headache groups and the NC group to explore the varying functional connectivity in the different subtypes of headache. Lastly, between-groups analysis was performed among the different subtypes of headache to explore the altered pattern of functional connectivity.

## Method

### Subjects

One hundred and ten subjects were recruited, including 18 EM patients, 16 CM patients, 44 MOH + CM patients, and 32 NCs. Patients were recruited from the International Headache Center, Department of Neurology, Chinese PLA General Hospital. All the following inclusion criteria should be fulfilled: 1) diagnosis of 1.3 CM, 8.2 MOH, and 1.1 and 1.2 migraine based on the International Classification of Headache Disorders, third Edition (beta version) (ICHD-III beta) [19]; 2) no migraine preventive medication used in the past 3 months; 3) age between 20 and 60 years; 4) right-handed; 5) absence of any chronic disorders, including hypertension, hypercholesterolemia, diabetes mellitus, cardiovascular diseases, cerebrovascular disorders, neoplastic diseases, infectious diseases, connective tissue diseases, other subtypes of headache, chronic pain other than headache, severe anxiety or depression preceding the onset of headache, psychiatric diseases, etc.; 6) absence of alcohol, nicotine, or other substance abuse; and 7) patient’s willingness to engage in the study. NCs were recruited from the hospital’s staff and their relatives. Inclusion criteria were similar to those of patients, except for the first two items. NCs should never have had any primary headache disorders or other types of headache in the past year. General demographic and headache information were registered and evaluated in our headache database. Additionally, we evaluated anxiety, depression, and cognitive function of all the participants by using the Hamilton Anxiety Scale (HAMA) [20], the Hamilton Depression Scale (HAMD) [21], the Chinese version of Mini-Mental State Examination (MMSE), and the Montreal Cognitive Assessment (MoCA) Beijing Version (www.mocatest.org). The exclusion criteria were the following: cranium trauma, illness interfering with central nervous system function, psychotic disorder, and regular use of a psychoactive or hormone medication. The study protocol was approved by the Ethical Committee of Chinese PLA General Hospital and complied with the Declaration of Helsinki. Informed consent was obtained from all participants before the study. MRI scans were taken in the interictal stage at least 3 days after a migraine attack for EM patients. All the subjects were right-handed and underwent conventional MRI examination to exclude the subjects with cerebral infarction, malacia, or occupying lesions. Alcohol, nicotine, caffeine, and other substances were avoided for at least 12 h before MRI examination.

### MRI acquisition

Images were acquired on a GE 3.0T MR system (DISCOVERY MR750, GE Healthcare, Milwaukee, WI, USA) and a conventional eight-channel quadrature head coil was used. All subjects were instructed to lie in a supine position, and formed padding was used to limit head movement. Conventional T2-weighted image (TR = 5000 ms, TE = 113.4 ms, FOV = 24 cm × 24 cm, Matrix = 384 × 384) and T1-FLAIR (TR = 2040 ms, TE = 6.9 ms, FOV = 24 cm × 24 cm, Matrix = 384 × 256) were obtained first. Then, the rs-fMRI was performed, during which subjects were instructed to relax, keep their eyes closed, stay awake, remain still, and clear their heads of all thoughts. Functional images were obtained using a gradient echo-planar imaging (EPI) sequence (TR = 2000 ms, TE = 30 ms, flip angle = 90, slice thickness = 3 mm, slice gap = 1 mm, FOV = 24 cm × 24 cm, Matrix = 64 × 64), and 180 continuous EPI functional volumes were acquired axially over 6 min. Finally, a high resolution three-dimensional T1-weighted fast spoiled gradient recalled echo (3D T1-FSPGR) sequence was performed, which generated 360 contiguous axial slices [TR (repetition time) = 6.3 ms, TE (echo time) = 2.8 ms, flip angle = 15°, FOV (field of view) = 25.6 cm × 25.6 cm, Matrix = 256 × 256, slice thickness = 1 mm]. None of the subjects complained of any discomfort or fell asleep during scanning. No obvious structural damage was observed based on the conventional MR images.

### Data processing

All MR structural and functional images were processed using Statistical Parametric Mapping 8 (SPM8) (http://www.fil.ion.ucl.ac.uk/spm) and the rs-fMRI data analysis toolkit (REST v1.8) [22] running under MATLAB 7.6 (The Mathworks, Natick, MA, USA).

The data preprocessing was carried out as follows: (1) The first ten volumes of each functional time course was discarded to allow for T1 equilibrium and the participants to adapt; (2) Slice timing; (3) Head motion correction; (4) Spatial normalization. These steps were performed by SPM8. No subjects had head motion with more than 1.5 mm displacement in X, Y, and Z direction or 1.5^0^ of any angular motion throughout the course of the scanning. The linear trend removal and temporal band-pass filtering (0.01–0.08 Hz) was performed by REST [22].

**Fig. 1:**
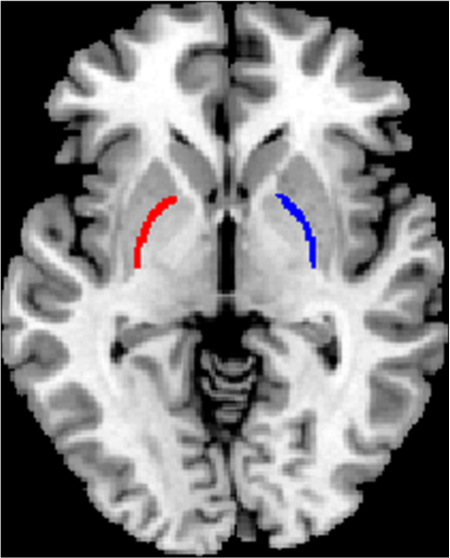
Bilateral marginal divison was created by manual drawing based on ch2bet template in MRIcron software

The functional connectivity analysis was performed as follows: (1) Spatial smooth (full width at half maximum (FWHM) = 6 mm) using SPM8; (2) MrD was defined using manual delineation on a *ch2bet* template in MRIcron software (v6.6, www.mricro.com) (Fig. 1); (3) Functional connectivity computation of the left and right MrD were performed using REST (v1.8). The time courses of bilateral MrD were extracted, and Pearson’s correlations were used to calculate the functional connectivity between the extracted time courses and the averaged time courses of the whole brain in a voxel-wise manner. The white matter, cerebrospinal fluid (CSF), and the six head motion parameters were used as covariates. (4) The individual r-maps were normalized to Z-maps using Fisher’s Z-transformation.

### Statistical analysis

One-way analysis of variance (ANOVA) was applied to the comparison of the age, BMI, education, migraine duration, headache frequency, pain intensity, medication intake, HAMA, HAMD, MMSE, and MoCA score. An independent sample t-test was applied to the comparison of the duration of headache chronicity/medication between the CM and MOH + CM groups. Significant difference was set at a *P* value of < 0.05. The statistical analysis was performed using SPSS 19.0.

One-sample t-tests were performed using the functional connectivity Z-maps to detect the regions with significant functional connectivity of MrD. Analysis of covariance (ANCOVA) tests were performed to identify the regions with significant differences in connectivity to MrD among groups, covarying for age, gender, and education. Significance was set at a *P* value of <0.001 without correction. The minimal number of contiguous voxels was set at 10. The statistical analysis was performed by SPM 8 software.

## Results

### Demography and neuropsychological test

Demographic and clinical data are summarized in Table 1. One hundred and ten subjects were included in our study, comprising 44 patients with MOH + CM (35 females, nine males, mean age 42.3 ± 9.6 years), 16 patients with CM (12 females, four males, mean age 42.4 ± 8.7 years), 18 patients with EM (14 females, four males, mean age 37.8 ± 7.9 years), and 32 NCs (24 females, eight males, mean age 41.3 ± 10.8 years). Although the age of the EM group tended to be lower than that of the other three groups, there was no significant difference for gender and age among groups. Body mass index (BMI) was significantly higher in the CM group than that of the EM and NC groups, and it was also higher in the MOH + CM group than in the EM group, using ANCOVA analysis with age as the covariance. The education level of the MOH + CM and CM groups was significantly lower than that of the EM and NC groups. We defined education in 6 levels from illiterate to master or higher as grades 1 to 6.

**Table 1:** Demographic information of the subjects (mean ± SD)

	EM	CM	MOH + CM	NC	*p* value
*N (F/M)*	18 (14/4)	16 (12/4)	44 (35/9)	32 (24/8)	0.97
Age (years)	33.4 ± 11.0**	42.4 ± 8.7	42.3 ± 9.6	41.3 ± 10.8	0.01
Body mass index	21.3 ± 0.7	24.4 ± 0.7*	23.1 ± 0.4	22.5 ± 0.5	0.01
Education	4.4 ± 1.1	3.4 ± 1.3**	3.1 ± 1.2**	4.7 ± 1.0	0.00
Migraine duration (years)	12.4 ± 8.1	11.3 ± 9.3	17.8 ± 9.1*	-	0.02
Headache frequency (days/month)	3.5 ± 2.7	25.1 ± 5.9**	26.5 ± 5.0**	-	0.00
Pain intensity (VAS)	8.3 ± 1.5	7.9 ± 1.5	8.0 ± 1.4	-	0.46
Duration of headache chronicity/medication overuse (years)	-	3.0 ± 3.3	4.7 ± 4.8	-	0.20
Medication intake	4.9 ± 3.7	4.3 ± 3.8	119.7 ± 111.6**	-	0.00
HAMA	15.7 ± 9.9**	21.6 ± 10.9**	18.5 ± 8.7**	2.4 ± 1.5	0.00
HAMD	10.9 ± 7.3**	16.3 ± 10.5**	19.9 ± 11.9**	1.1 ± 0.9	0.00
MMSE (adjusted by education)	28.5 ± 0.7	26.9 ± 0.7	27.1 ± 0.5	28.3 ± 0.6	0.22
MoCA (adjusted by education)	26.1 ± 0.8	23.5 ± 0.9^**^	24.8 ± 0.6 ^*^	26.9 ± 0.7	0.02

* *VAS* visual anologue score, *HAMA* Hamilton anxiety scale, *HAMD* Hamilton depression scale, *MMSE* mini-mental state examination, *MoCA* the Montreal cognitive assessment, *EM* episodic migraine, *CM* chronic migraine, *MOH + CM* medication overuse headache plus with chronic migraine *:compared to NCs *P <* 0.05;**:compared to NCs *P* < 0.01

The mean years of migraine was significantly higher in the MOH + CM group (mean 17.8 ± 9.1 years) than that in the CM (11.3 ± 9.3 years) and EM (12.4 ± 8.1 years) groups. Headache frequency was significantly higher in the MOH + CM (mean headache days per month 26.5 ± 5.0) and CM (25.1 ± 5.9) groups than that in the EM group (3.5 ± 2.7). There was no significant difference in chronic headache duration between the MOH + CM group and the CM group or in pain intensity between the patient groups. The MOH + CM group took much more medication (mean number of pills per month 119.7 ± 111.6) than the CM (4.3 ± 3.8) and EM (4.9 ± 3.7) groups. The types of overused medication by MOH + CM patients included simple analgesics (3/44), triptan (1/44), opioids (1/44), combination analgesics (33/44), and multiple drug classes (6/44). CM patients and EM patients most frequently took combination analgesics as painkiller. None of the migraine patients regularly took preventive medication during the past 3 months.

Anxiety and depression scores were significantly higher in the three headache groups than that in NC group. The MOH + CM group showed a higher depression score, and the CM group showed a higher anxiety score than the EM group (*P* < 0.05). Cognitive function showed no significant difference among groups evaluated by MMSE but declined in the MOH + CM (mean score 24.8 ± 0.6) and CM (23.5 ± 0.9) groups compared with the NC group when evaluated by MoCA.

### Functional connectivity of MrD - within-group analysis

Within-group analysis was performed, and a false discovery rate (FDR) was used with a *p* value set at < 0.05 with an extended threshold of 10 voxels. Regions with connectivity to MrD in each of the groups were acquired, and the functional connectivity maps were marked on the SPM8 T1 template.

**Fig. 2:**
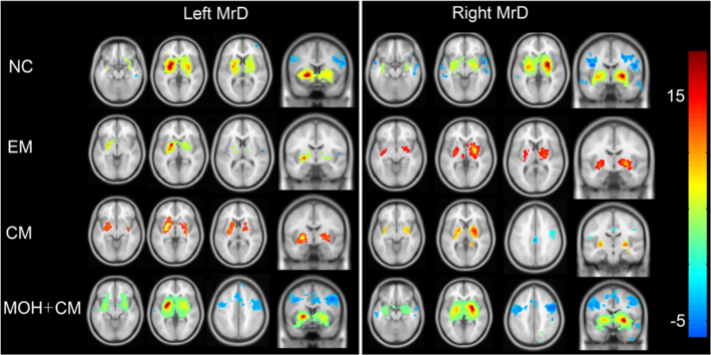
MrD functional connectivity averaged over subject in the brain. Warm and cool colors represent positive and negative correlations. NC, normal control; EM, episodic migraine; CM, chronic migraine; MOH+CM, medication overuse headache plus chronic migraine;L, *left* MrD; R, *right* MrD

Regions with positive functional connectivity of MrD were mainly in the bilateral basal ganglion regions, thalamus, insula, hippocampus, and right medial frontal orbital cortex, and the regions of negative functional connectivity of MrD were in the bilateral temporal lobes and middle frontal lobes in the NC group (Fig. 2).

In the EM group, regions with positive functional connectivity were mainly located in bilateral basal ganglion regions, and no evident negative functional connectivity was observed (Fig. 2).

In the CM and MOH + CM groups, regions with positive functional connectivity were located in bilateral basal ganglion regions. The regions with negative functional connectivity in the MOH + CM group were larger compared with the CM group, which were located in the bilateral middle frontal gyrus, cingulum, and right temporal lobes (Fig. 2).

### Comparison of functional connectivity of MrD between the migraine groups and NC group

**Fig. 3:**
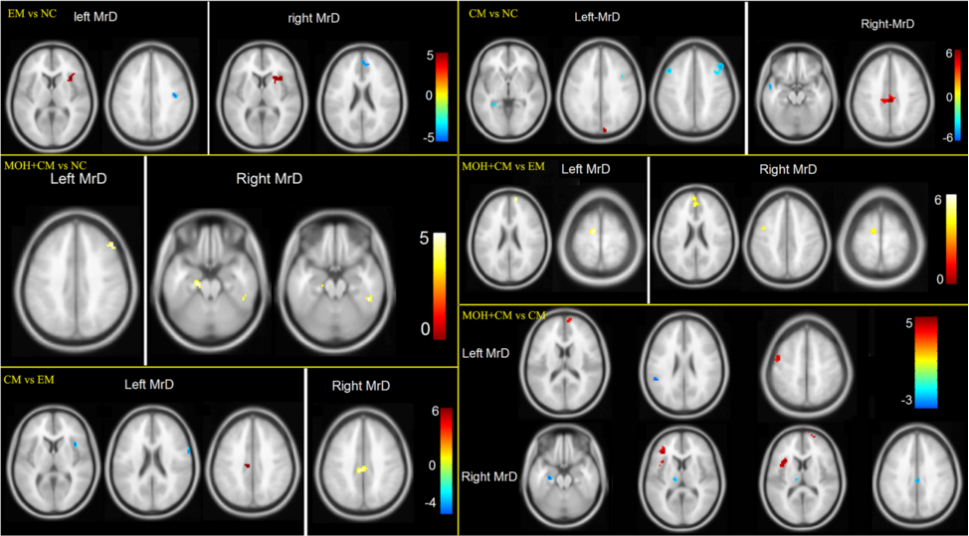
Comparison of MrD functional connectivity among subjects group. Warm color represents decreased MrD functional connectivity in EM compared with NC, and cool color represent increased MrD functional connectivity. NC, normal control; EM, episodic migraine; CM, chronic migraine; MOH+CM, medication overuse headache plus chronic migraine; MrD, marginal division

Table 2 shows the altered functional connectivity of MrD in migraineurs compared with NCs. In the EM group, the brain regions with decreased functional connectivity were mainly located in the right insula, and the brain regions with increased functional connectivity were mainly located in the right precentral gyrus and anterior cingulate cortex (ACC) (Fig. 3). In the CM group, the decreased functional connectivity of MrD was observed in the right cuneus and left middle cingulum cortex (MCC), and the increased functional connectivity was detected in the bilateral middle frontal gyrus, left hippocampus, and middle temporal gyrus (Fig. 3).

**Table 2:** Comparison of functional connectivity of MrD between headache group and HC group

Group	Brain region	k value	*P* value	T value	x	y	z
EM vs. NC							
EM<NC							
Left	Insula_R	27	0.000	4.2	36	21	6
Right	Insula_R	23	0.000	4.3	33	18	6
EM>NC							
Left	Precentral_R	13	0.000	4.5	42	−15	36
Right	Cingulum_Ant_R	17	0.000	4.4	12	45	21
CM vs. NC							
CM<NC							
Left	Cuneus_R	10	0.000	4.7	9	−90	30
Right	Cingulum_Mid_L	66	0.000	5.7	0	−27	39
CM>NC							
Left	Frontal_Mid_L	21	0.000	4.52	−45	15	45
	Hippocampus_L	12	0.000	4.06	−30	−39	0
	Frontal_Mid_R	51	0.000	4.05	48	24	42
Right	Temporal_Mid_L	15	0.000	4.35	−54	3	−21
MOH + CMvs. NC							
MOH + CM>NC							
Left	Frontal_Mid_R	10	0.000	3.87	48	30	39
Right	Temporal_inf_R	12	0.000	4.56	48	−39	−21
	ParaHippocampa_L	18	0.000	4.42	−18	−21	−24

*R* right hemisphere, *L* left hemisphere, *Ant* anterior, *Mid* middle

Interestingly, the decreased functional connectivity of MrD could not be observed in the MOH + CM group, while the increased functional connectivity was demonstrated in the left parahippocampus, right middle frontal gyrus, and inferior temporal gyrus (Fig. 3).

### Comparison of functional connectivity of MrD among the CM, MOH + CM, and EM groups

Table 3 presents the altered functional connectivity of MrD among the CM, MOH + CM, and EM groups. Decreased functional connectivity of MrD was detected in the left middle cingulum, and increased functional connectivity was observed in the right insula and precentral gyrus in the CM group compared with the EM group (Fig. 3).

**Table 3:** Comparison of functional connectivity of MrD among CM group, MOH + CM group and EM group

Groups	Brain region	k value	*P* value	T value	x	y	z
CM vs. EM							
CM<EM							
Left	Cingulum_Mid_L	10	0.000	3.9	−9	−24	39
Right	Cingulum_Mid_L	42	0.000	5.0	−6	−27	39
CM>EM							
Left	Insula_R	17	0.000	5.0	39	15	6
	Precentral_R	12	0.000	4.7	66	3	21
MOH + CM vs.EM							
MOH + CM<EM							
Left	Frontal_Sup_Media_R	15	0.000	4.4	12	60	24
	Supp_Motor_Area_L	13	0.000	4.3	−12	−9	69
Right	Frontal_Sup_Media_L	53	0.000	5.1	−3	60	18
	Supp_Motor_Area_L	24	0.000	4.1	−9	−12	66
	Precentral_L	12	0.000	3.8	−39	0	45
MOH + CMvs.CM							
MOH + CM<CM							
Left	Precentral_L	19	0.000	4.9	−45	0	51
	Frontal_Sup_Media_R	11	0.000	4.4	9	60	18
Right	Frontal_Inf_Tri_L	16	0.000	4.8	−36	33	9
	Insula_L	42	0.000	4.6	−33	18	12
	Frontal_Sup_R	10	0.000	3.8	21	63	12
MOH + CM>CM							
Left	Temporal_Sup_L	10	0.000	3.9	−48	−42	24
Right	Hippocampus_L	13	0.000	4.8	−27	−18	−18
	Cingulum_Mid_R	14	0.000	4.1	3	−27	33
	Thalamus_L	10	0.000	3.7	−15	−18	9

*R* right hemisphere, *L* left hemisphere, *Ant* anterior, *Mid* middle, *Media* medial, *Sup* superior, *Inf* inferior, *Tri* triangular part

In the MOH + CM group, decreased functional connectivity of MrD was demonstrated in the bilateral medial superior frontal gyrus, left precentral gyrus, and supplementary motor area compared with the EM group (Fig. 3). No increased functional connectivity of MrD was observed.

**Fig. 4:**
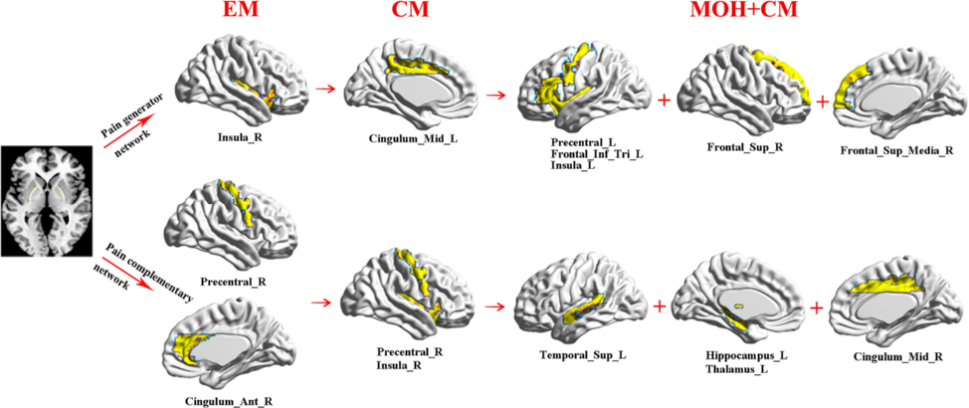
Double neuromodulation network of MrD including three order pain generator network and three order pain complementary networkᅟ

The decreased functional connectivity of MrD was detected in the left precentral gyrus, the triangular part of the inferior frontal gyrus, the insula, the right medial superior frontal gyrus, and the superior frontal gyrus, and increased functional connectivity was observed in the left superior temporal gyrus, hippocampus, thalamus, and right middle cingulum in the MOH + CM group compared with the CM group (Fig. 3, 4).

## Discussion

Migraines are a common type of primary headaches with a reported prevalence of approximately 5.7 % in men and 17.0 % in women [23]. In China, the prevalence of migraine is 9.3 % of the general population [24]. Migraines are also a major cause of chronic headaches, with approximately 2.5 % of EM transformed to new-onset CM [25]. The prevalence rate of CM is 2 to 4 % of the general population [25], and that of MOH is 1 to 4 % of the general population [26]. Therefore, chronicization of migraine is a worthy topic of further investigation.

The brain regions related with pain processing and modulation mainly included the prefrontal cortex, basal ganglia, thalamus, cingulate cortex, insula, cerebellum [27], and periaqueductal gray matter [28]. In this study, MrD was investigated to reveal its key roles in migraine chronicization using rs-fMRI.

Functional connectivity is actually the correlation analysis between the brain regions with MrD over the whole brain. The normal brain structure includes positive and negative functional connectivity to maintain the brain’s functional balance. Altered functional connectivity may indicate the intrinsic pathophysiological changes for different brain disorders.

In this study, it was demonstrated that there was functional connectivity between other brain regions and MrD in the NCs and the patients with migraine. The normal connectivity pattern, such the positive associations with the bilateral basal ganglion nuclei, thalamus, insula, hippocampus, and medial frontal orbital cortex, and the negative associations with bilateral temporal lobes and middle frontal lobes, indicated that MrD was an important subcortical center. Previous report referred to it as a subcortical memory center. Interestingly, we found that MrD was also a subcortical pain center due to positive and negative associations with multiple brain regions in different subtypes of headache. In this study, only the positive connectivity was demonstrated in the EM group, and both the positive and negative connectivities were confirmed in the CM and MOH + CM groups, which suggested that MrD demonstrated different pain modulation patterns in different subtypes of headache.

Between-groups analysis showed altered intrinsic functional connectivity in different subtypes of headache compared with NCs. The decreased functional connectivity of MrD was located in the right insula in the EM group, which was a component of the pain matrix. The insula seemed to play a leading role in the triggering of pain matrix network, and resulted in the subjective pain experience [29]. fMRI also demonstrated that the insula could process emotion and sensory-discriminative aspects of pain perception [30]. The impaired functional connectivity between MrD and the insula might disturb the balance of pain modulation of the insula in EM patients. Simultaneously, the functional connectivity was strengthened between the right precentral gyrus and the anterior cingulum cortex (ACC) and MrD. The increased connectivity could be understood as a positive feedback to maintain the concordance of the pain matrix network.

Compared with NCs, decreased functional connectivity was observed in the right cuneus and left middle cingulum in the CM group, which suggested that the cuneus and middle cingulum may be signature brain structures in chronic migraine, and may contribute to migraine chronicization. The cuneus is related to visual information processing [31], is responsible for the integration of the somatosensory information with other sensory stimuli and cognitive process [32], and could be activated with other pain-related brain regions [33]. The decreased functional connectivity between MrD and the cuneus may impair this integration, and facilitate migraine chronicization. The decreased functional connectivity between MrD and MCC was a new finding in CM patients compared previous studies [34–37] in which only the ACC was related to pain modulation, and the posterior cingulum cortex (PCC) was related to pain processing and cognitive impairment in migraineurs. The increased functional connectivity of MrD in the bilateral middle frontal gyrus, left hippocampus, and middle temporal gyrus indicated a dynamic compensation for the dysfunction of the pain-related brain regions in CM patients.

Only increased functional connectivity was confirmed in the left parahippocampus and right middle frontal and inferior temporal gyrus in the MOH + CM group, which indicated that the dysfunction of MrD could not be detected. The reasons for this may be explained as follows: (1) The negative functional connectivity of MrD was activated, and the impaired MrD network was compensated and strengthened; (2) Medication overuse may inhibit the modulation function of MrD and provide some protection for MrD. Therefore, the impairment of MrD connectivity was avoided. (3) The brain regions with increased functional connectivity may contribute to pain integration and protect the pain matrix.

Comparison of functional connectivity among different subtypes of headache demonstrated that MrD played a key role in migraine chronicization. The decreased functional connectivity of MrD could be detected from EM to CM, from EM to MOH + CM, and from CM to MOH + CM. Additionally, the increased FM could also be detected from EM to CM and from CM to MOH + CM. However, the increased functional connectivity was not revealed from EM to MOH + CM. The decreased connectivity pattern revealed the dysfunction of MrD, which could explain the role of MrD in migraine chronicization. Therefore, MrD can be regarded as a subcortical pain center. The increased connectivity indicated that MrD could maintain the concordance of the pain matrix network.

On the basis of rs-fMRI findings, it can be speculated that MrD plays a double role in the neuromodulation of migraine (Fig. 4). One type of neuromodulation is the negative pain network, which includes three-order pain generating networks, and the functional connectivity is decreased among these networks. The first-order pain generating network is the right insula, which is the EM generator (EMG) and demonstrates decreased functional connectivity with MrD. The second-order pain generating network is the left middle cingulate cortex (MCC), which is the CM generator (CMG) and also demonstrates decreased functional connectivity with MrD. These findings also suggested that the left MCC played a key role in migraine chronicization. The last-order pain-generating network is the MOH + CM generator (MOHG). This order pain network mainly included two brain regions: (1) the right superior frontal gyrus and medial superior frontal gyrus; (2) the left precentral gyrus, left pars triangularis of inferior frontal gyrus, and left insula.

The other neuromodulation is the positive pain network, which includes three-order pain complementary networks and the functional connectivity was strengthened among these networks. The first-order pain complementary network is located in the right precentral gyrus and right ACC, which may repair the dysfunction of MrD neuromodulation in EM patients. The second-order pain complementary network is mainly revealed in the right precentral gyrus and right insula, which could compensate for the dysfunction of MrD neuromodulation and prevent migraine chronicization. The last-order pain complementary network is involved in the left superior temporal gyrus, left hippocampus, left thalamus, and right MCC. These brain regions may improve the state of the pain network in MOH + CM patients.

The double neuromodulation network of MrD indicated that the three-order pain generating network and the three-order pain complementary network were the important neuromodulation patterns of MrD in migraines. The involved specific brain regions could be considered as target pain network biomarkers, and early-warning signals of neuromodulation in different subtypes of migraine.

There were some limitations in the present study. First, this study was a cross-sectional study, and the sample sizes of the EM and CM groups were relatively small. Future studies are needed to carry out longitudinal analysis to dynamically observe migraine chronicization and the real roles of MrD in this process. Second, task-based fMRIs should be performed to identify the key roles of MrD in pain modulation and transformation.

## Conclusions

 This study is the first to characterize the roles of MrD in the different subtypes of headache using rs-fMRI. The major findings are that the FC of MrD was demonstrated in the different subtypes of headache, and altered FC was revealed among different groups. These data indicated that MrD may play an important role in pain modulation and migraine chronicization, and the mechanism requires further investigation.

## Abbreviations

CM: Chronic migraine
EM: Episodic migraine
MOH: Medication overuse headache
MrD: Marginal division
NC: Normal control
